# Supplementary figures and images for: Plastid Phylogenomics of Paeonia and the Evolution of Ten Flower Types in Tree Peony
Source: Genes (Basel). 2022 Nov 27;13(12):2229. doi: 10.3390/genes13122229 (PMC9778541; doi:10.3390/genes13122229)

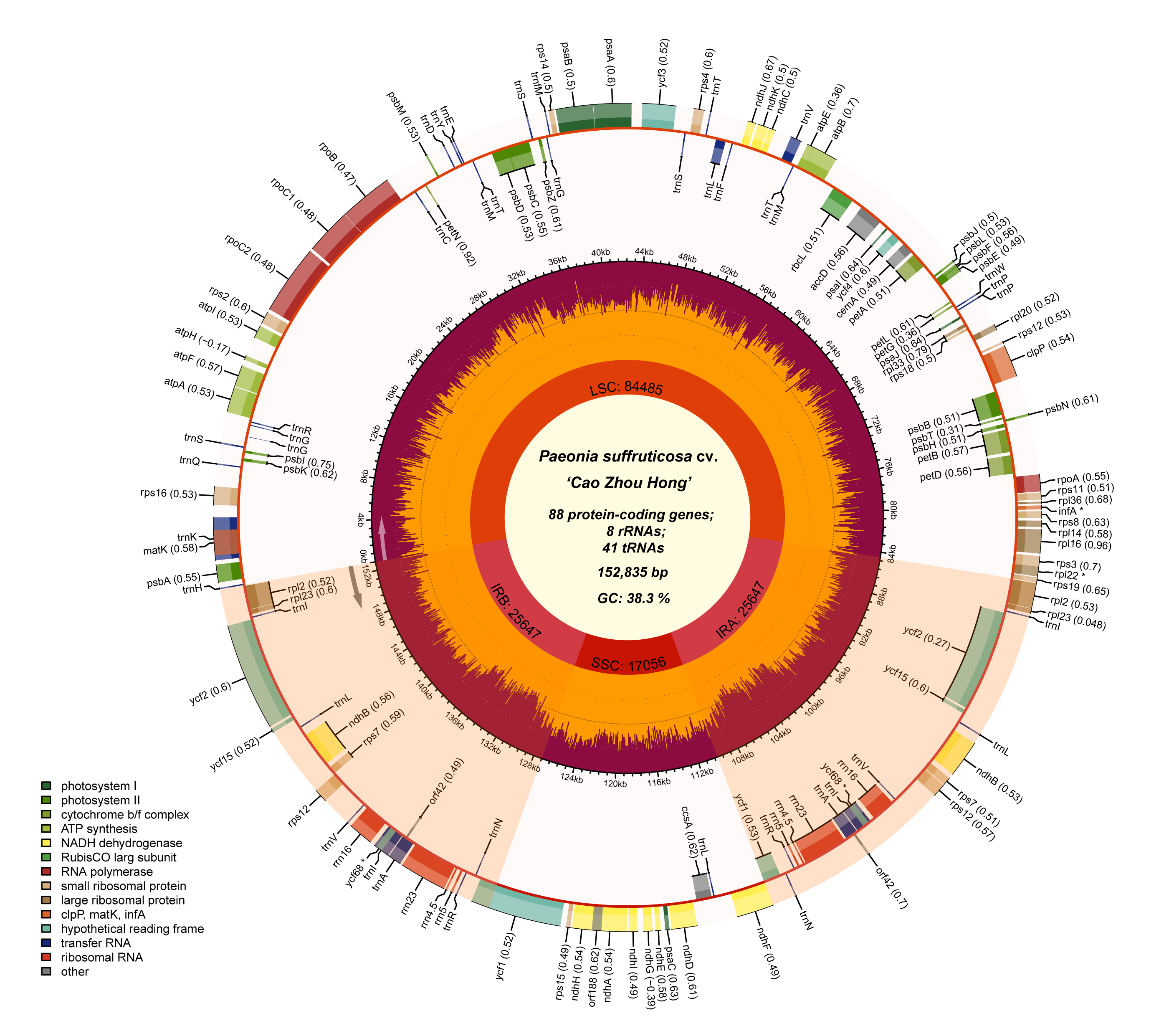

Supplement: Supplementary file 1 [file genes-13-02229-s001.zip › Supplementary Figure/Supplementary Figure S1.tif]

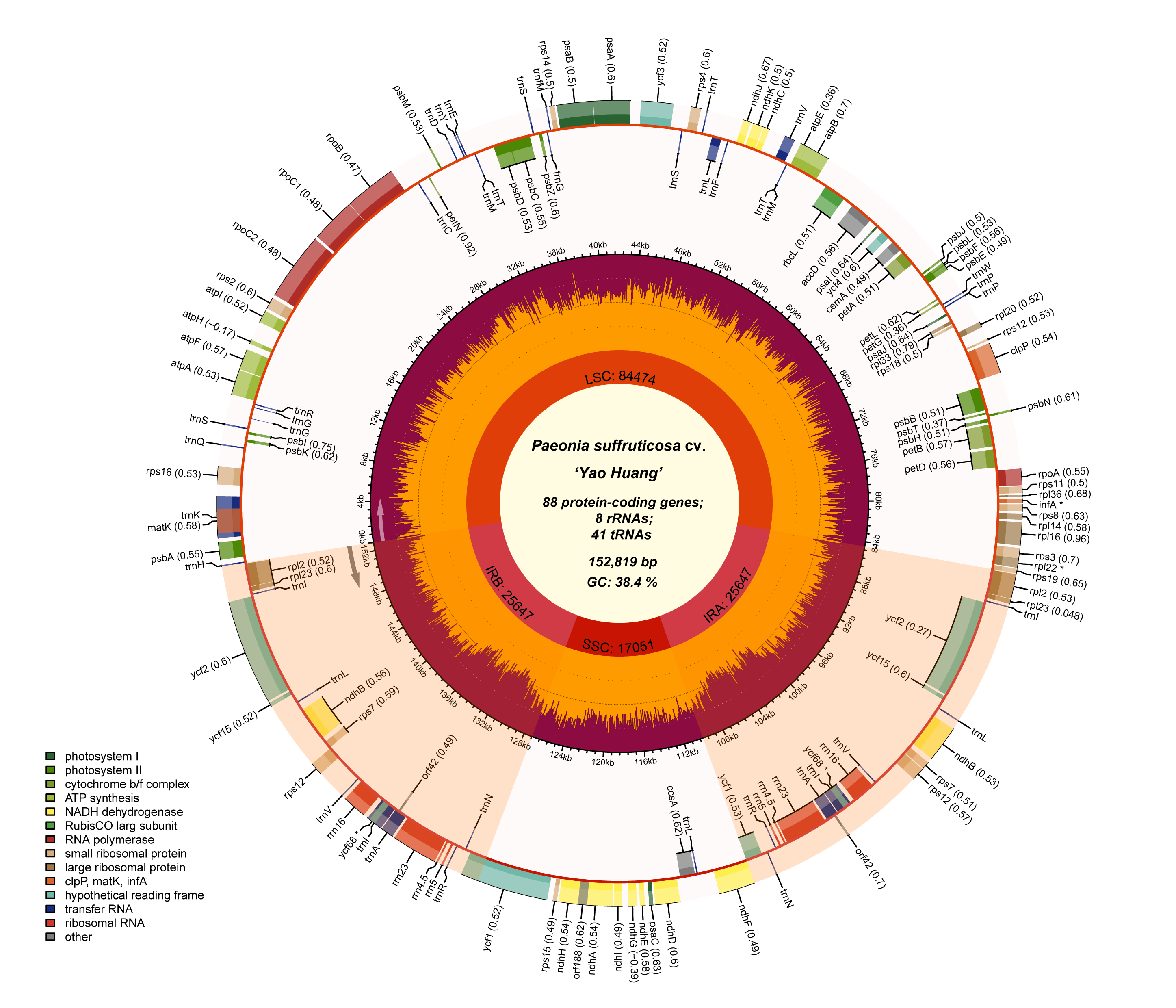

Supplement: Supplementary file 1 [file genes-13-02229-s001.zip › Supplementary Figure/Supplementary Figure S10.tif]

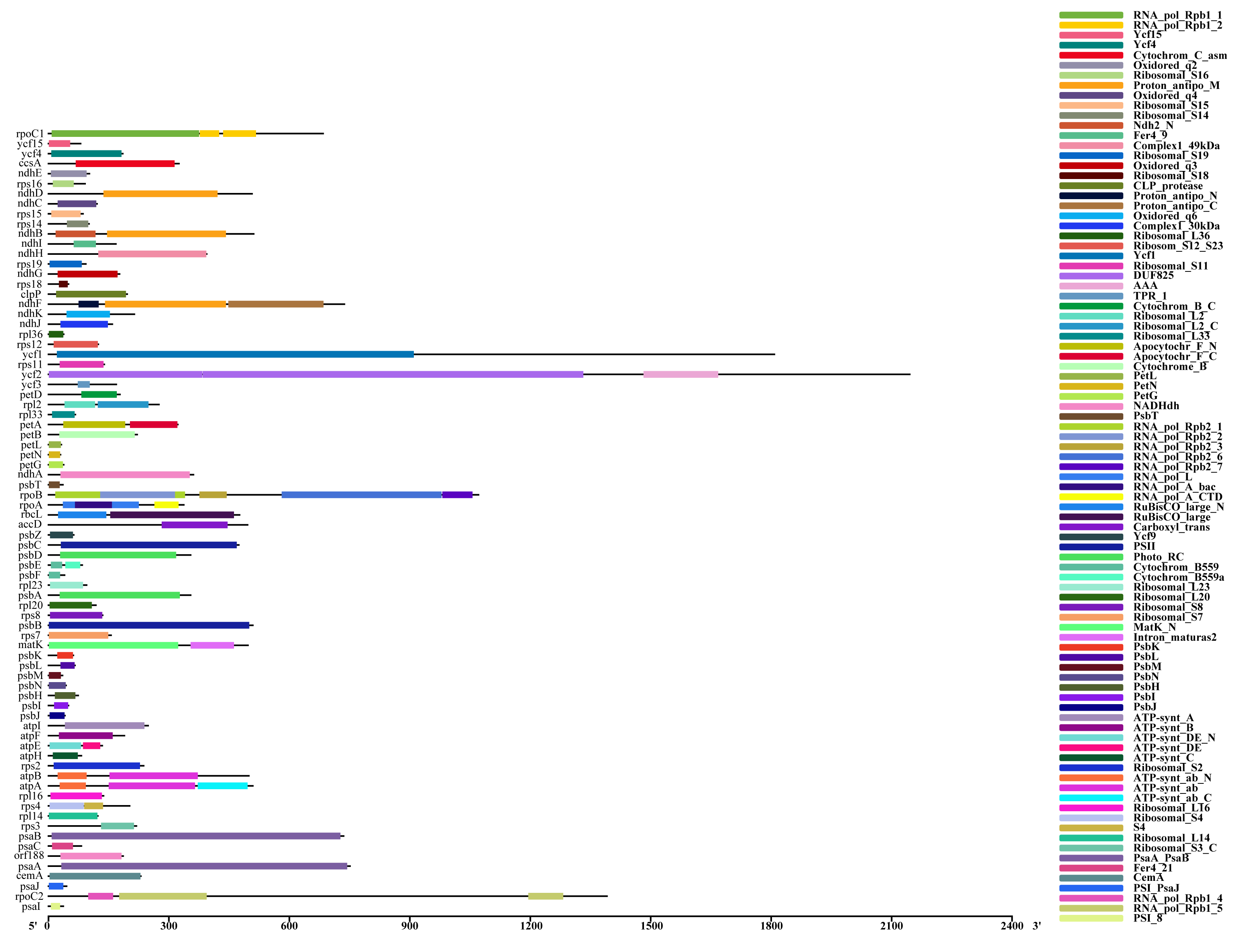

Supplement: Supplementary file 1 [file genes-13-02229-s001.zip › Supplementary Figure/Supplementary Figure S11.tif]

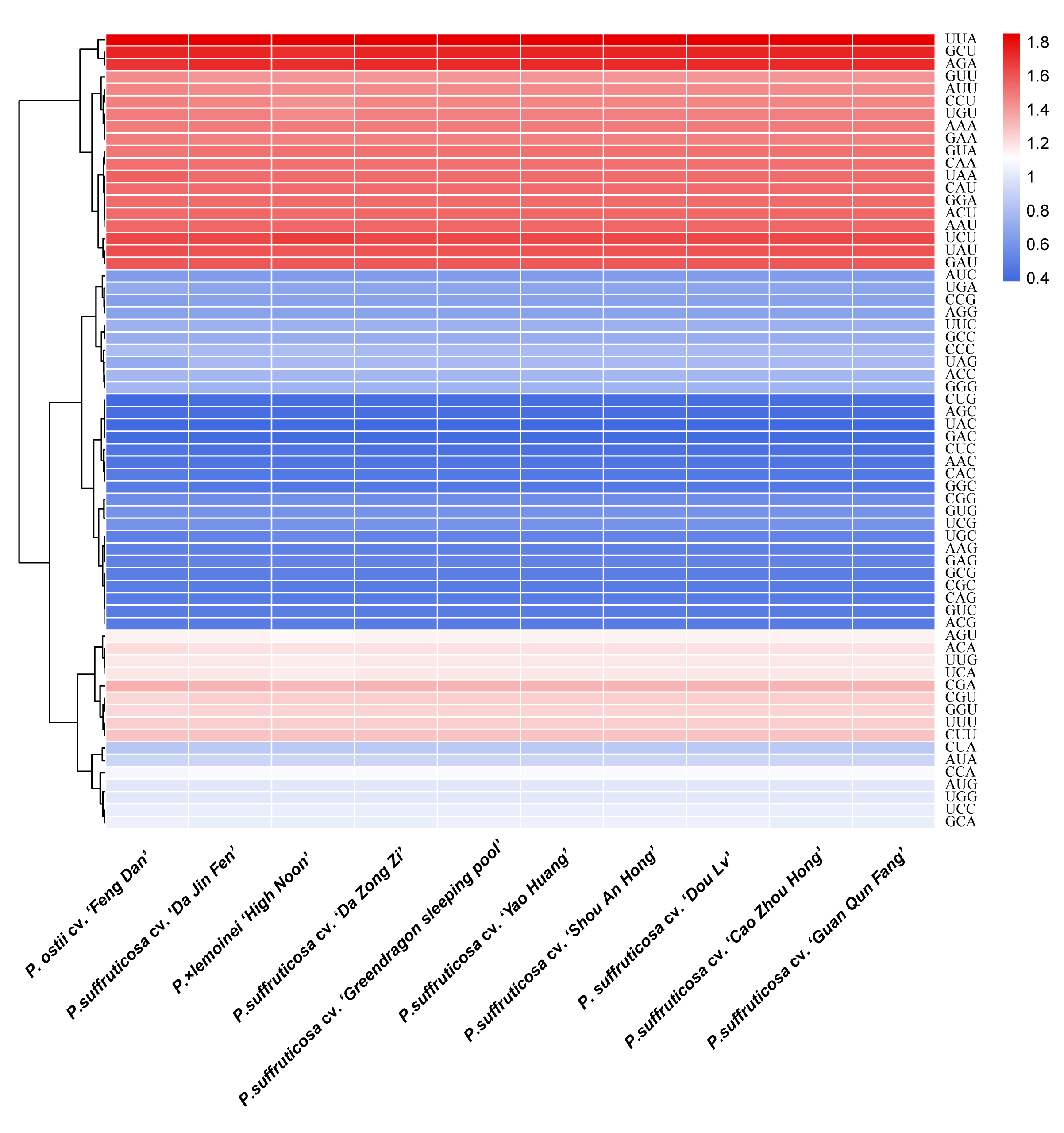

Supplement: Supplementary file 1 [file genes-13-02229-s001.zip › Supplementary Figure/Supplementary Figure S12.tif]

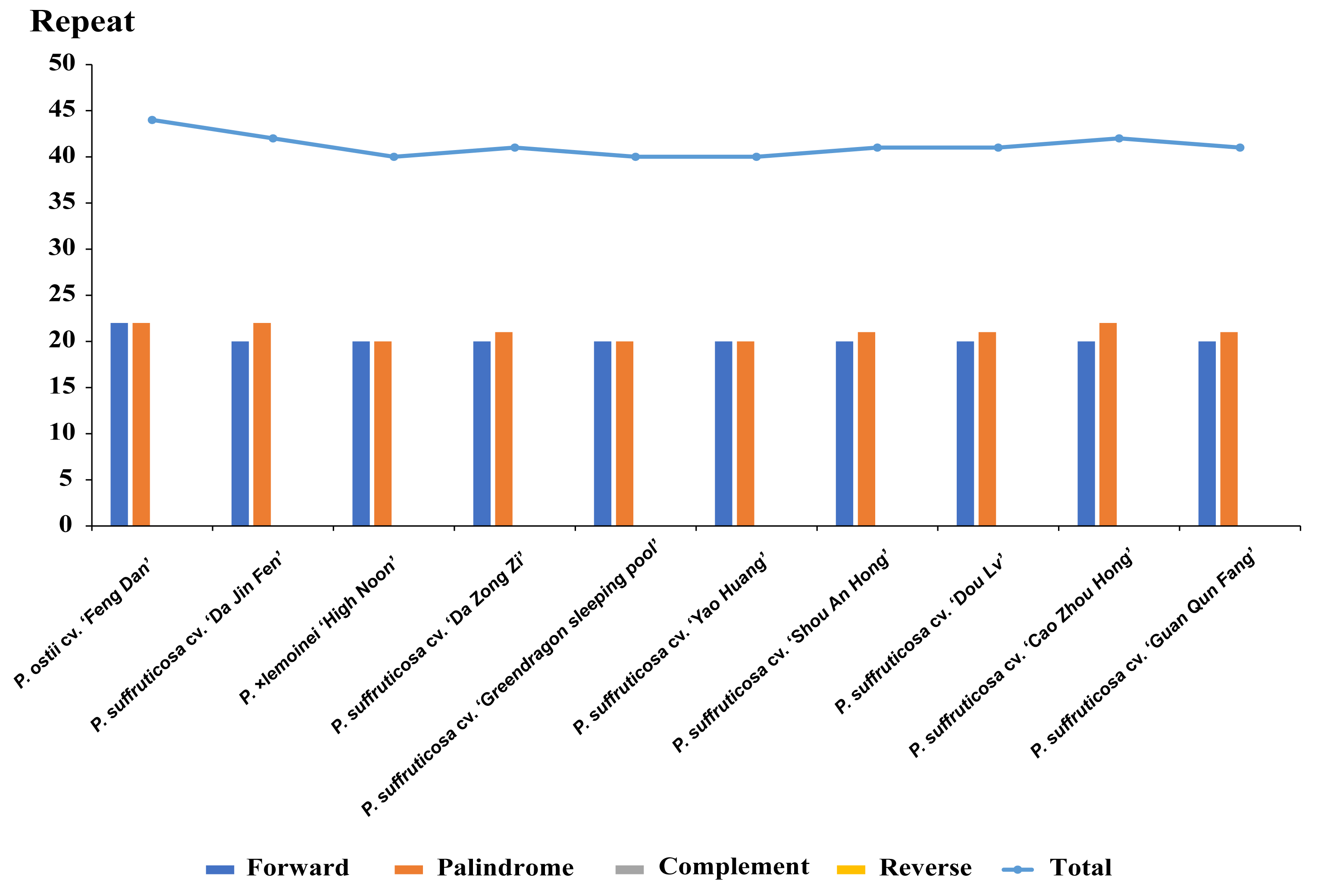

Supplement: Supplementary file 1 [file genes-13-02229-s001.zip › Supplementary Figure/Supplementary Figure S13.tif]

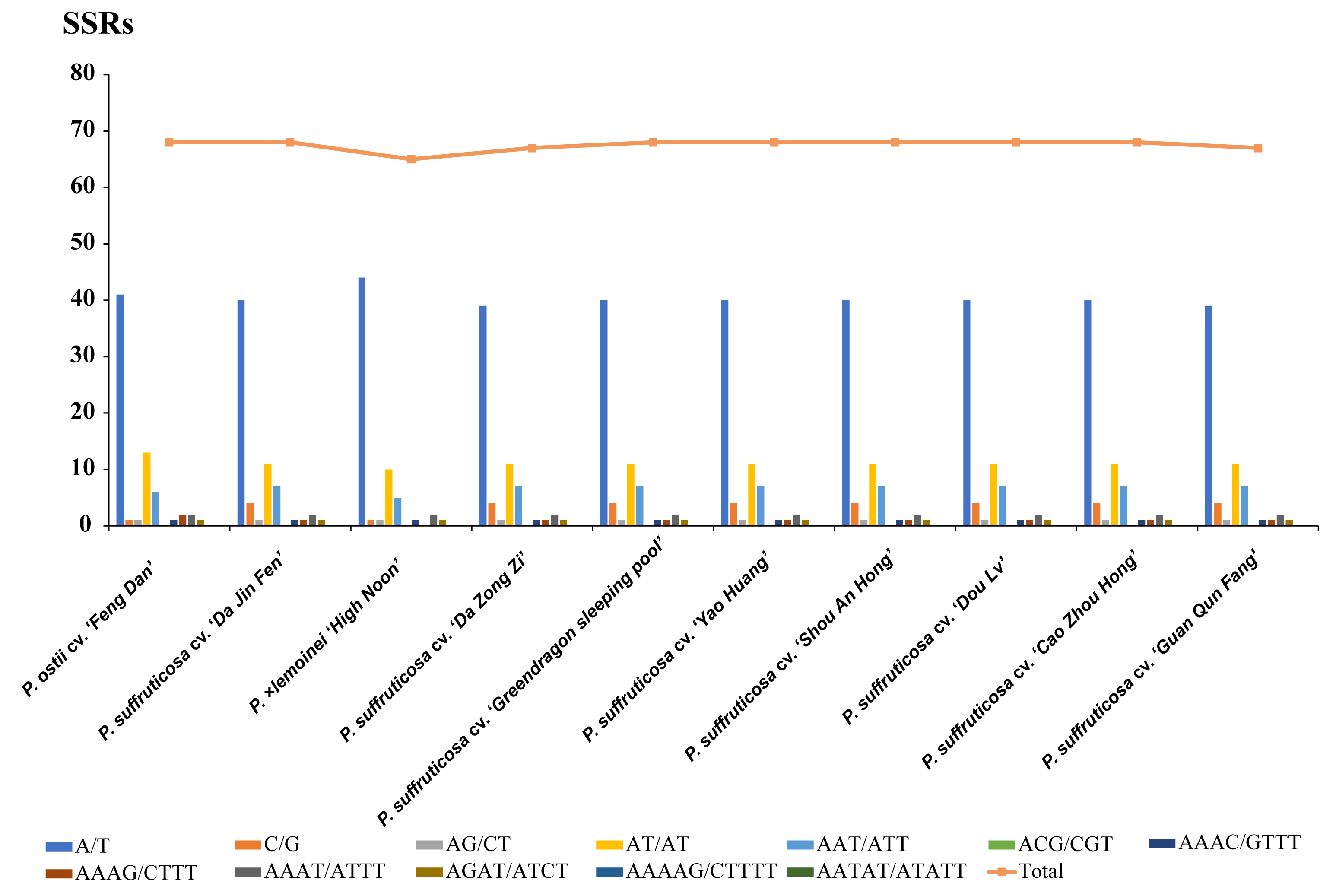

Supplement: Supplementary file 1 [file genes-13-02229-s001.zip › Supplementary Figure/Supplementary Figure S14.tif]

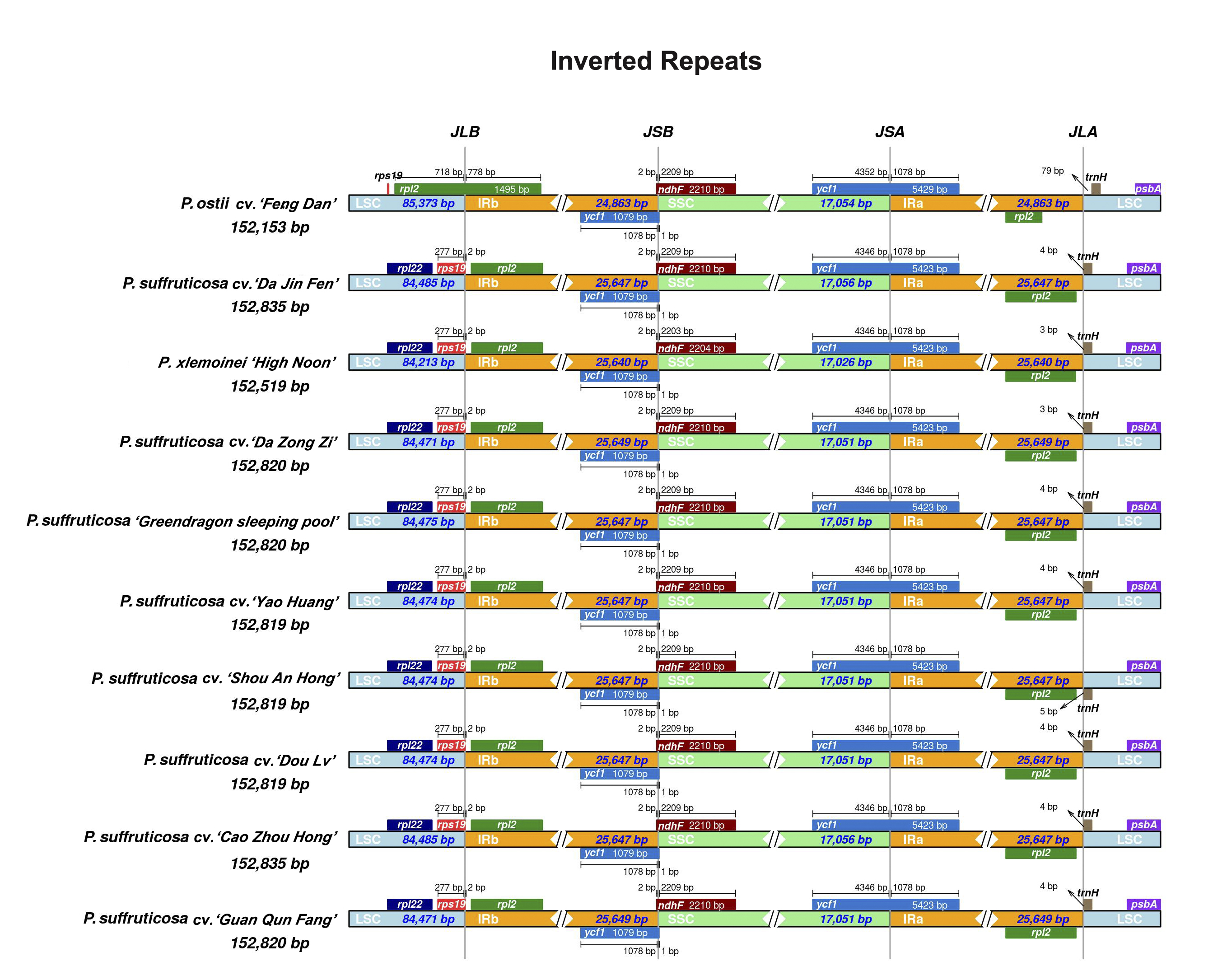

Supplement: Supplementary file 1 [file genes-13-02229-s001.zip › Supplementary Figure/Supplementary Figure S15.tif]

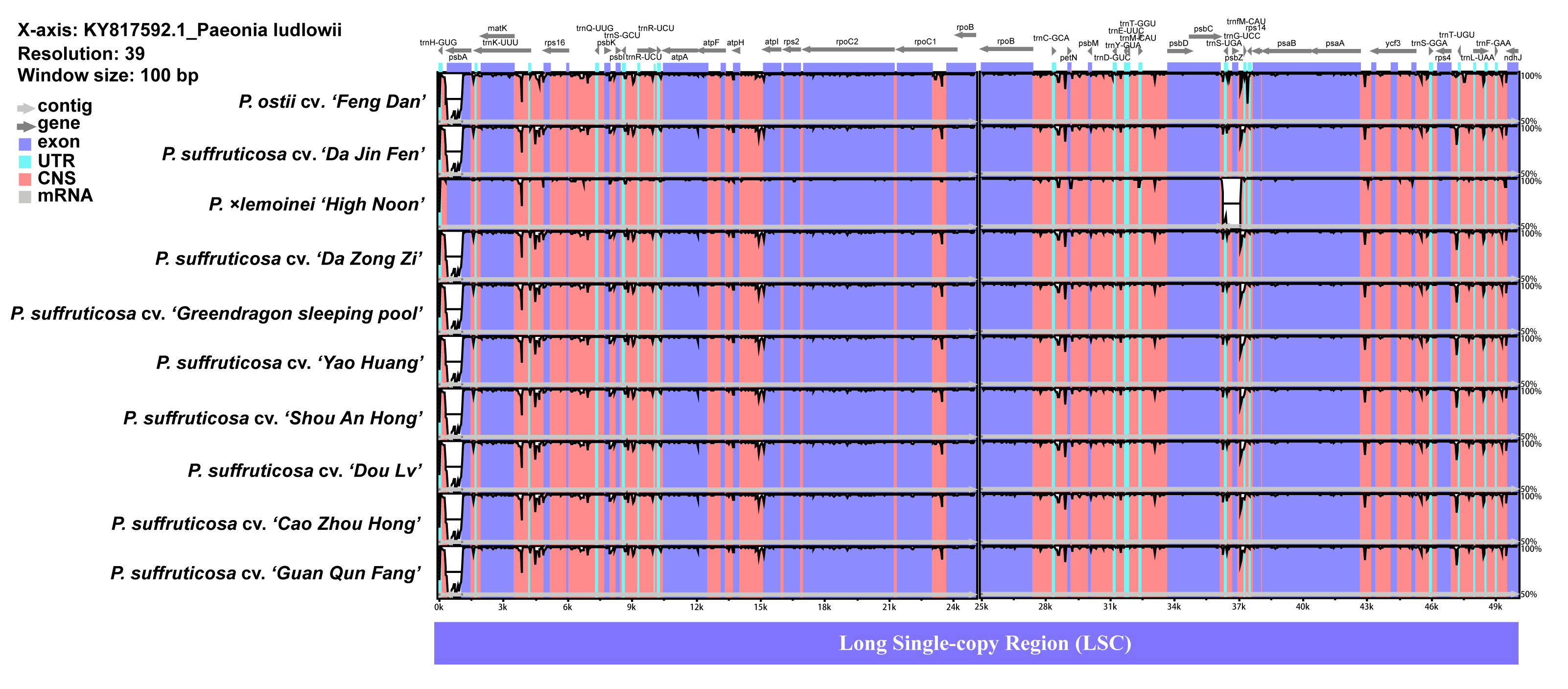

Supplement: Supplementary file 1 [file genes-13-02229-s001.zip › Supplementary Figure/Supplementary Figure S16(A).tif]

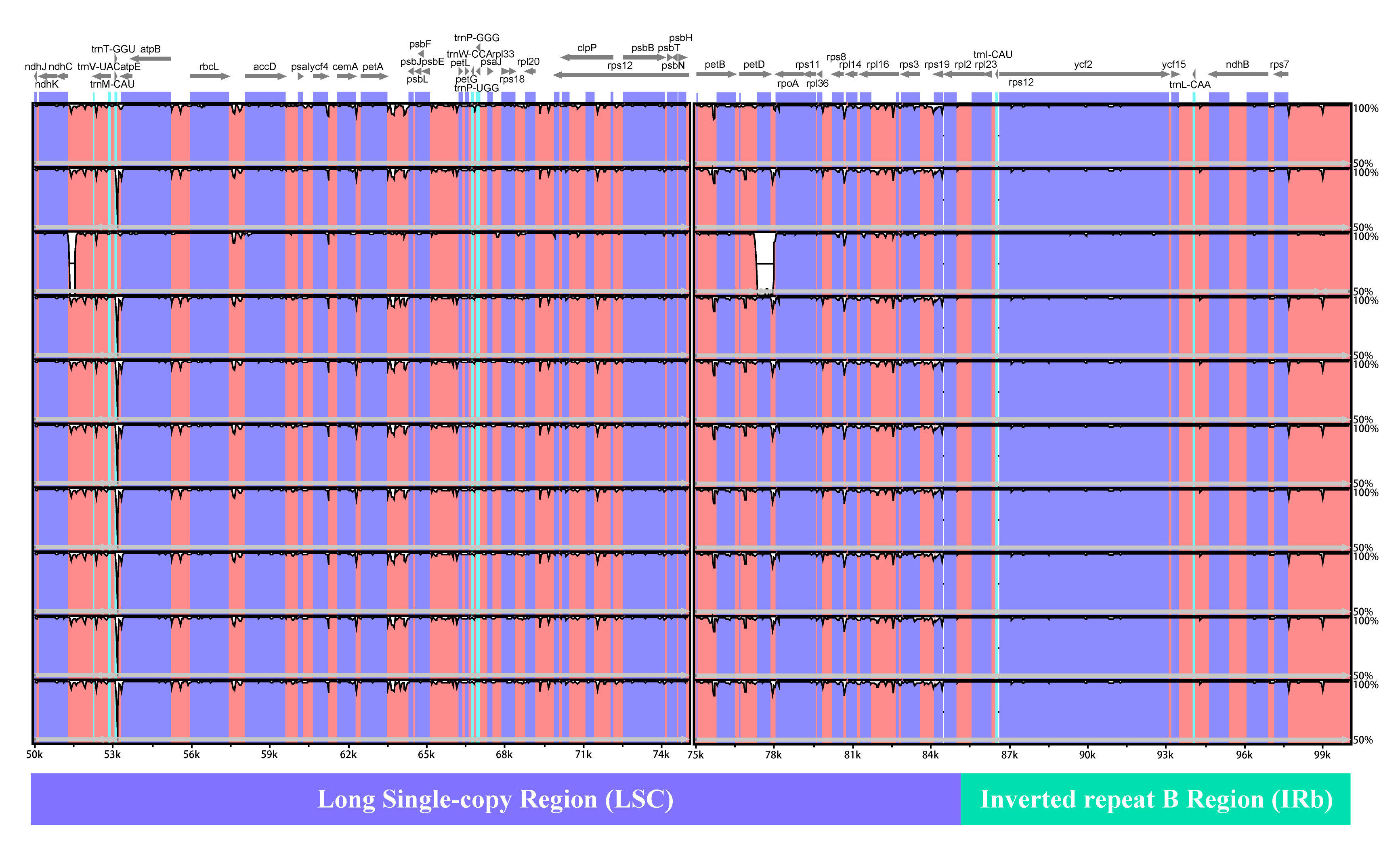

Supplement: Supplementary file 1 [file genes-13-02229-s001.zip › Supplementary Figure/Supplementary Figure S16(B).tif]

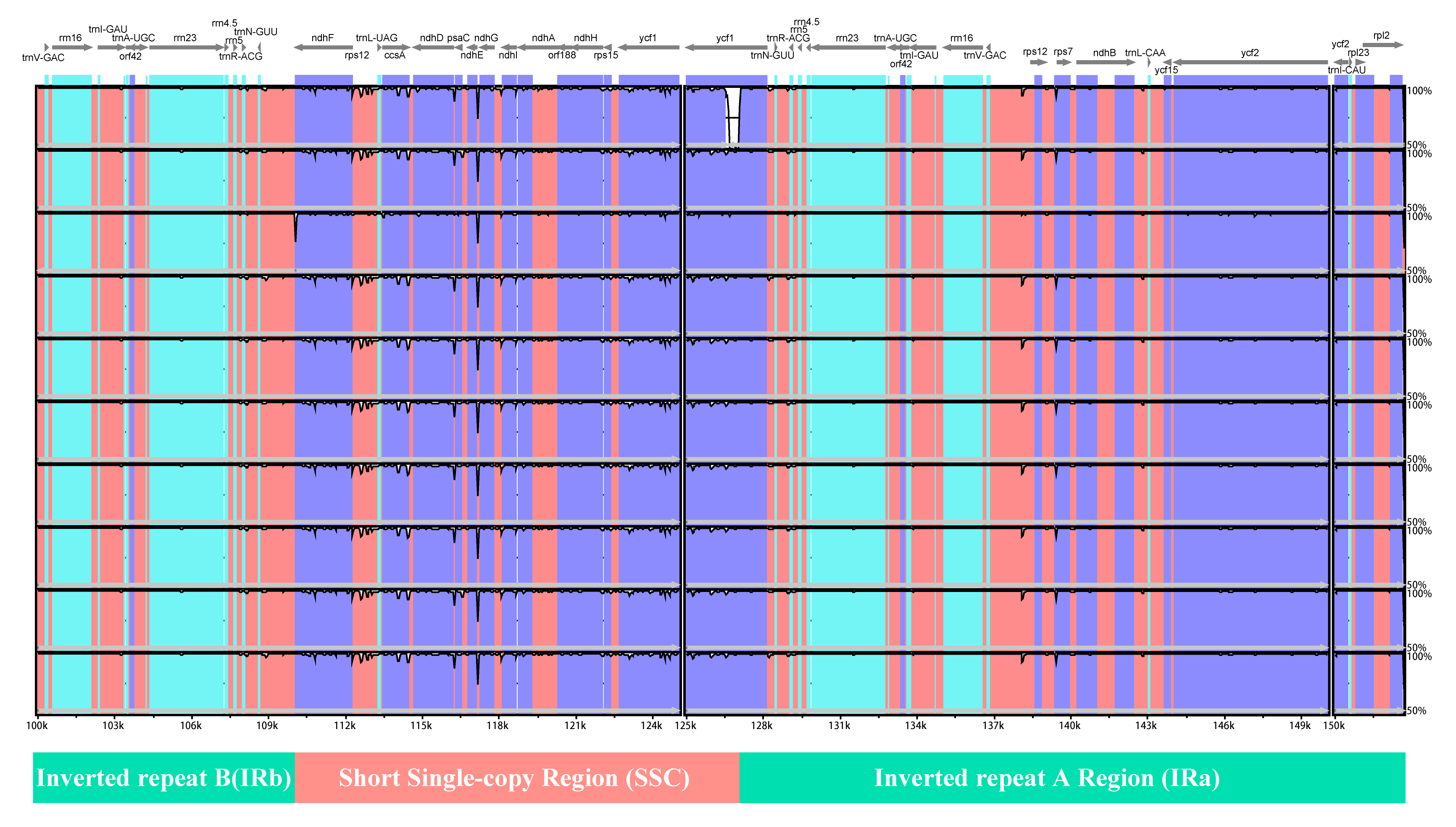

Supplement: Supplementary file 1 [file genes-13-02229-s001.zip › Supplementary Figure/Supplementary Figure S16(C).tif]

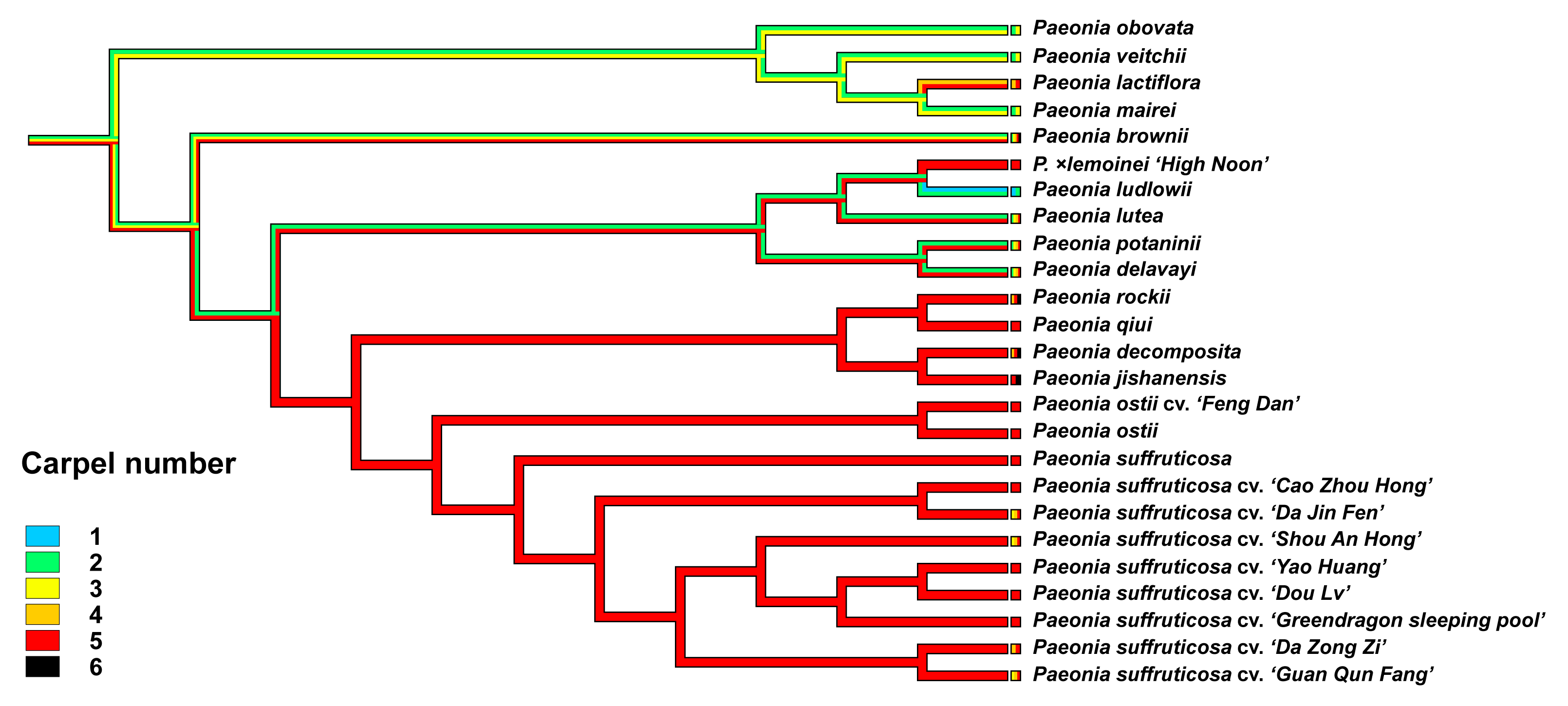

Supplement: Supplementary file 1 [file genes-13-02229-s001.zip › Supplementary Figure/Supplementary Figure S17.tif]

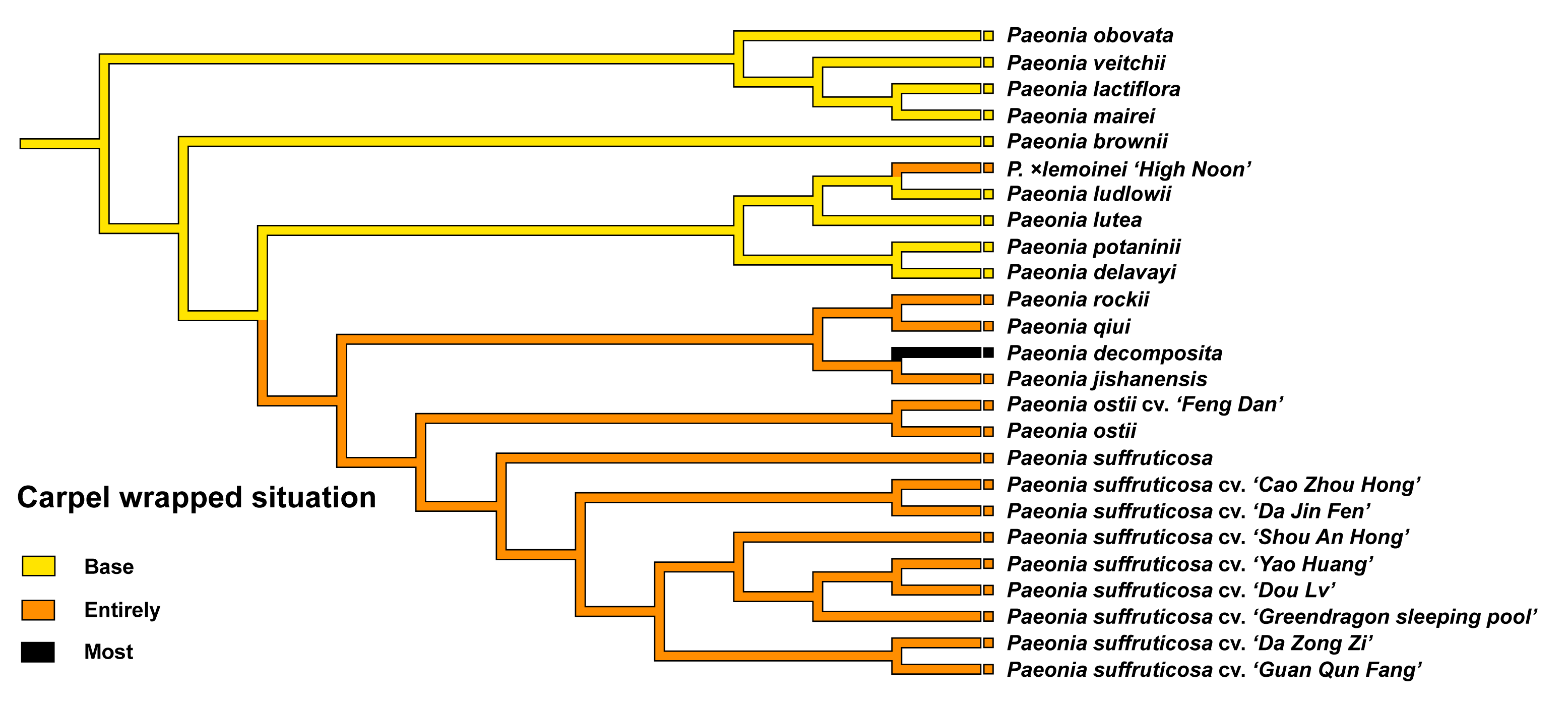

Supplement: Supplementary file 1 [file genes-13-02229-s001.zip › Supplementary Figure/Supplementary Figure S18.tif]

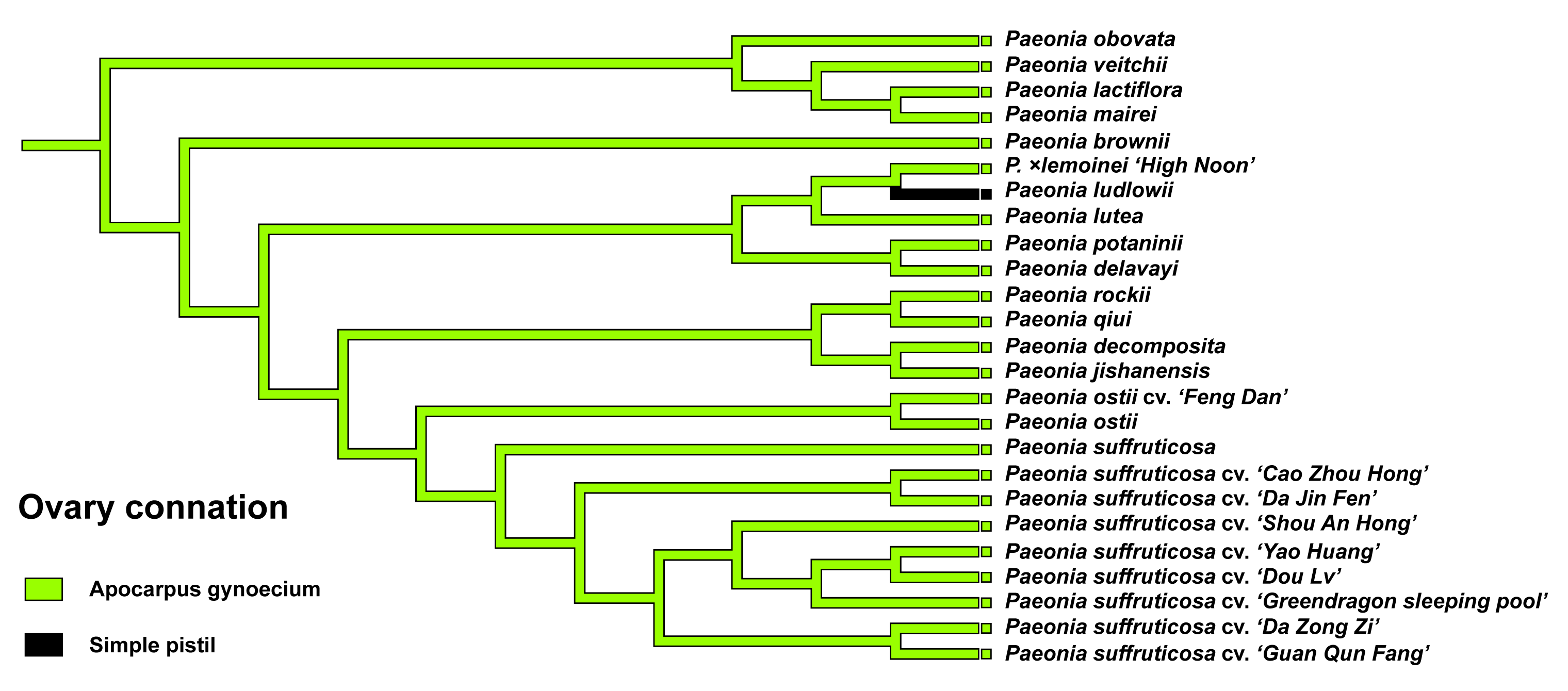

Supplement: Supplementary file 1 [file genes-13-02229-s001.zip › Supplementary Figure/Supplementary Figure S19.tif]

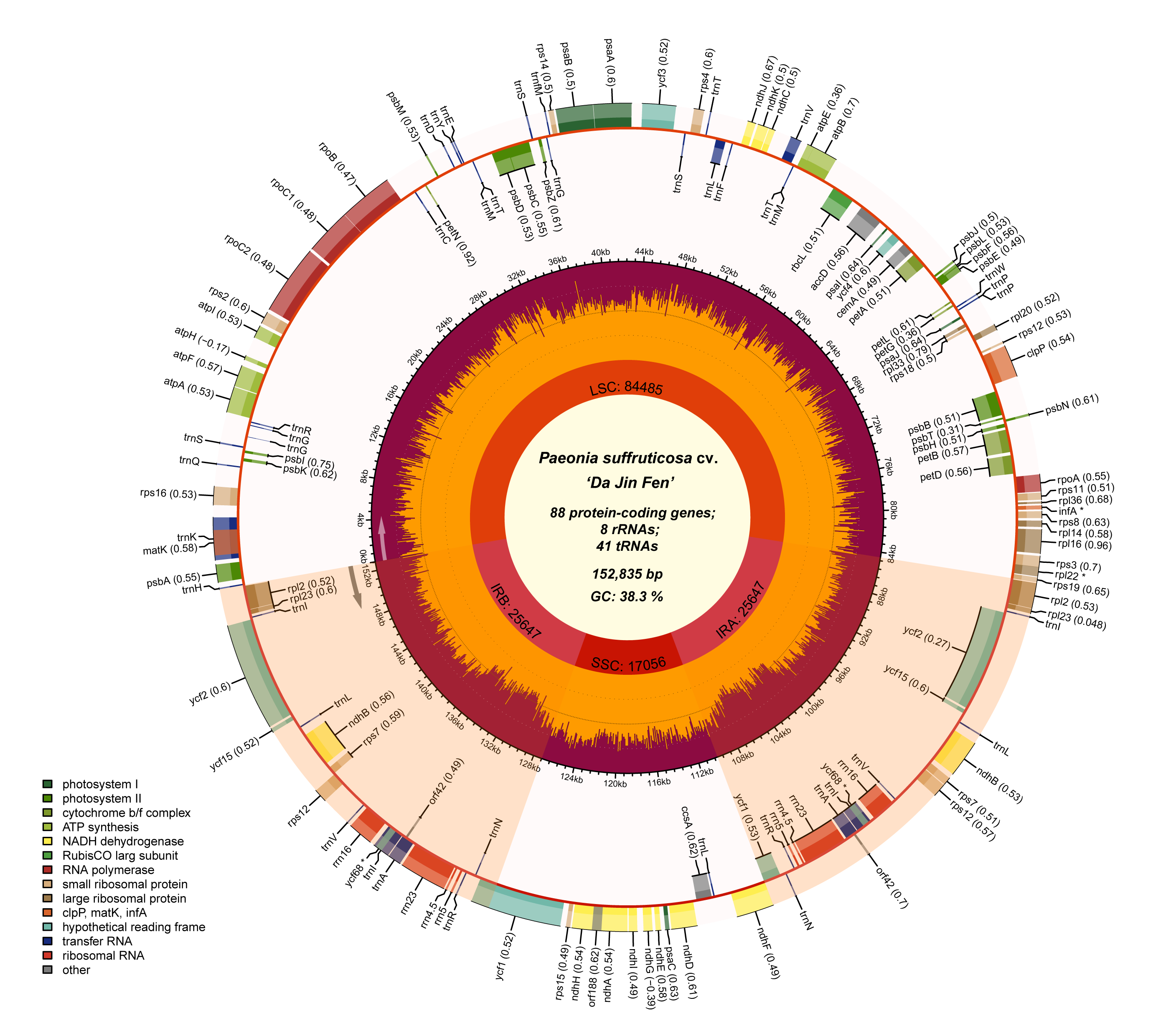

Supplement: Supplementary file 1 [file genes-13-02229-s001.zip › Supplementary Figure/Supplementary Figure S2.tif]

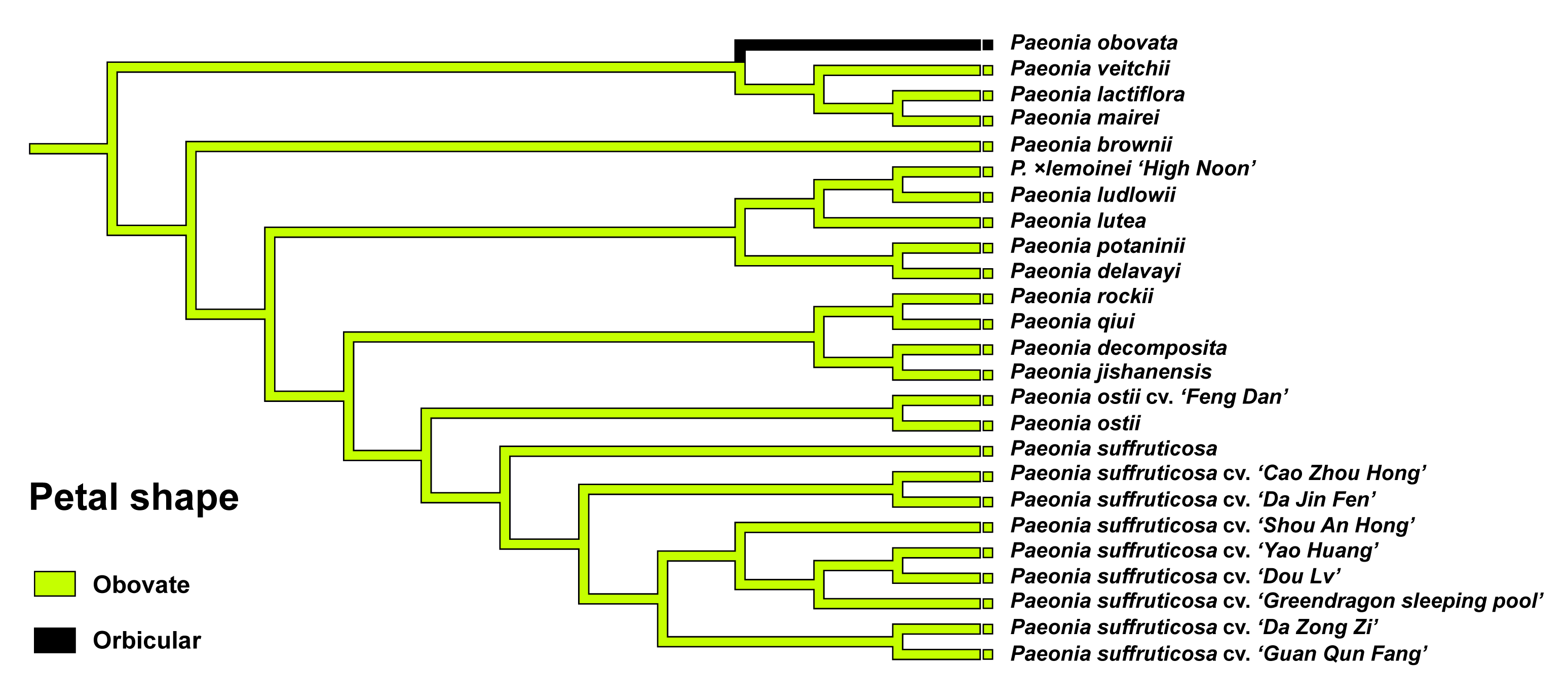

Supplement: Supplementary file 1 [file genes-13-02229-s001.zip › Supplementary Figure/Supplementary Figure S20.tif]

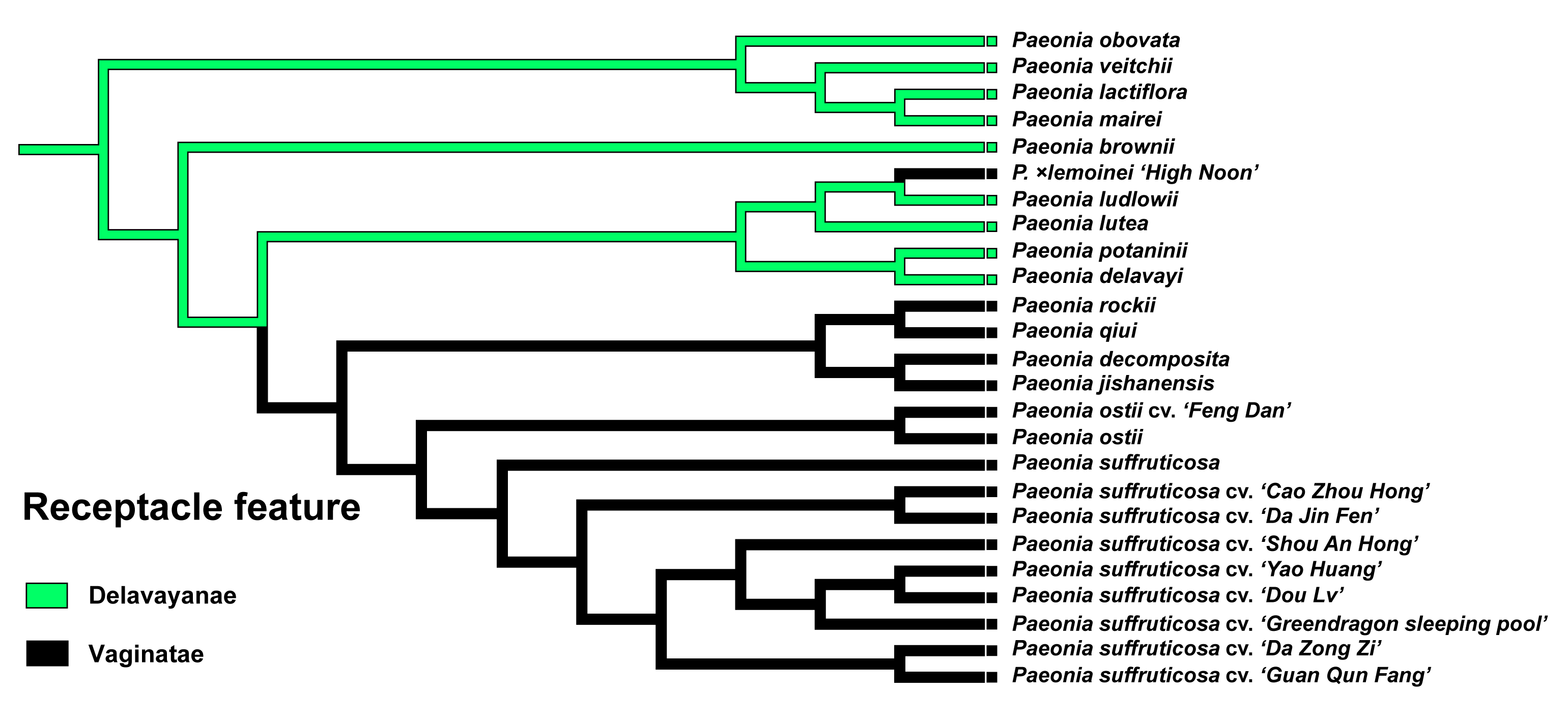

Supplement: Supplementary file 1 [file genes-13-02229-s001.zip › Supplementary Figure/Supplementary Figure S21.tif]

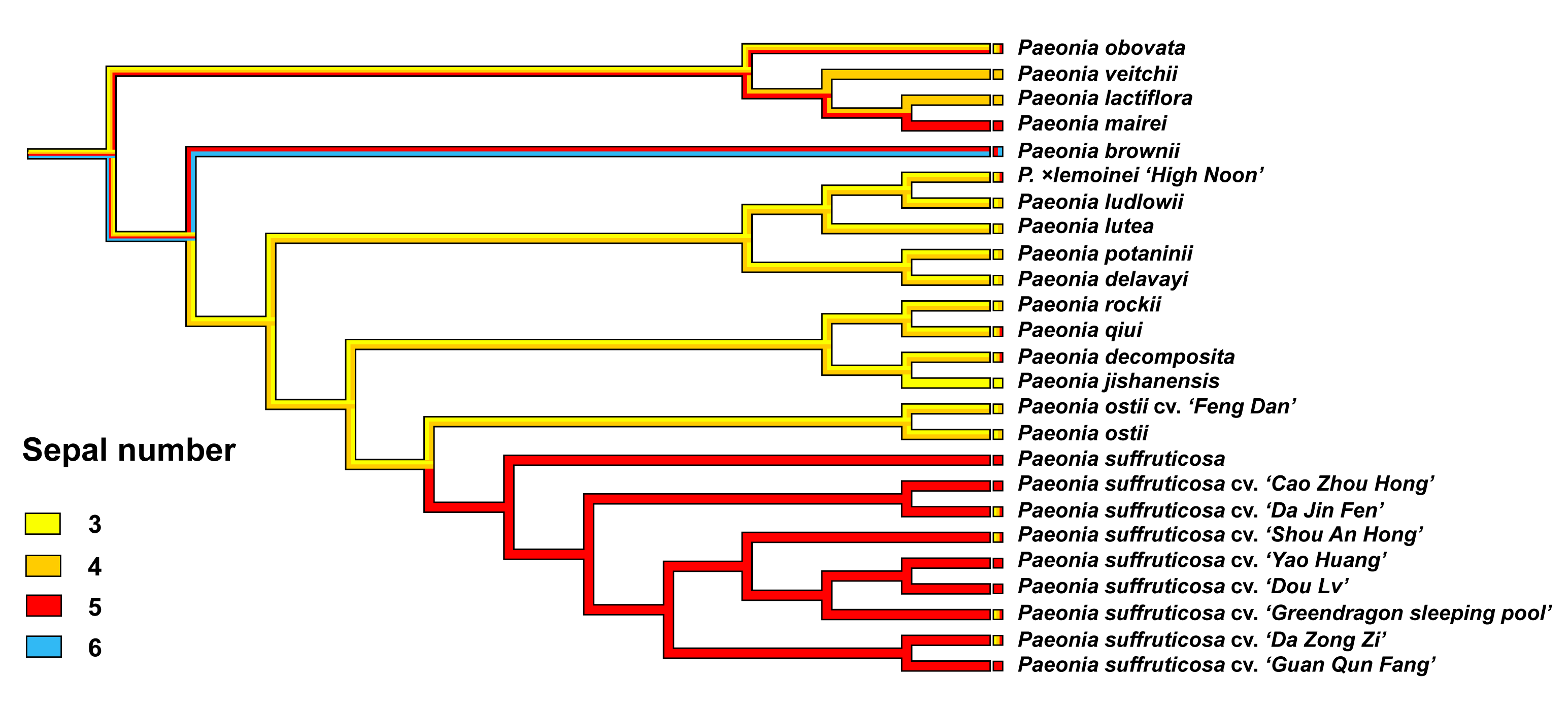

Supplement: Supplementary file 1 [file genes-13-02229-s001.zip › Supplementary Figure/Supplementary Figure S22.tif]

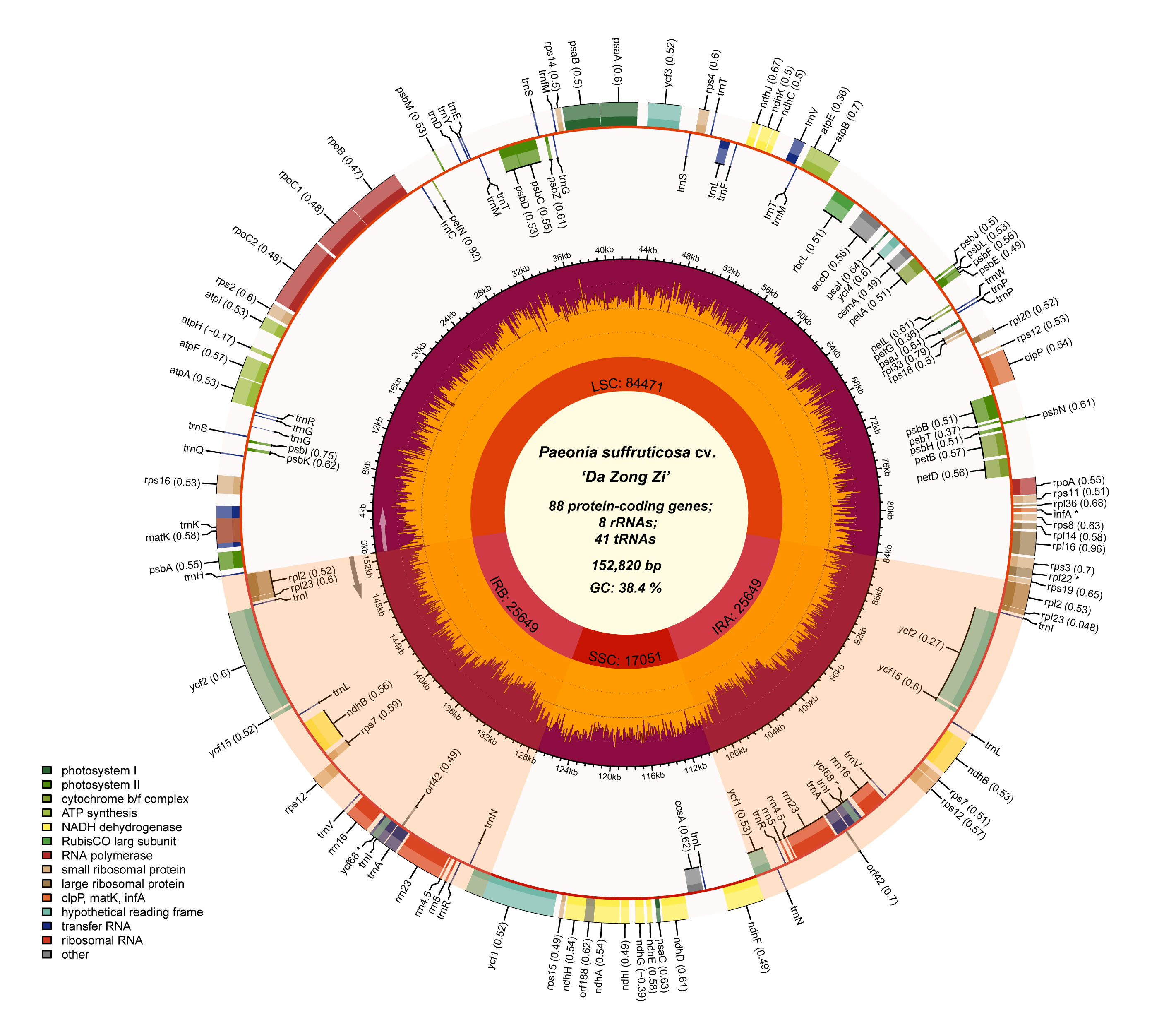

Supplement: Supplementary file 1 [file genes-13-02229-s001.zip › Supplementary Figure/Supplementary Figure S3.tif]

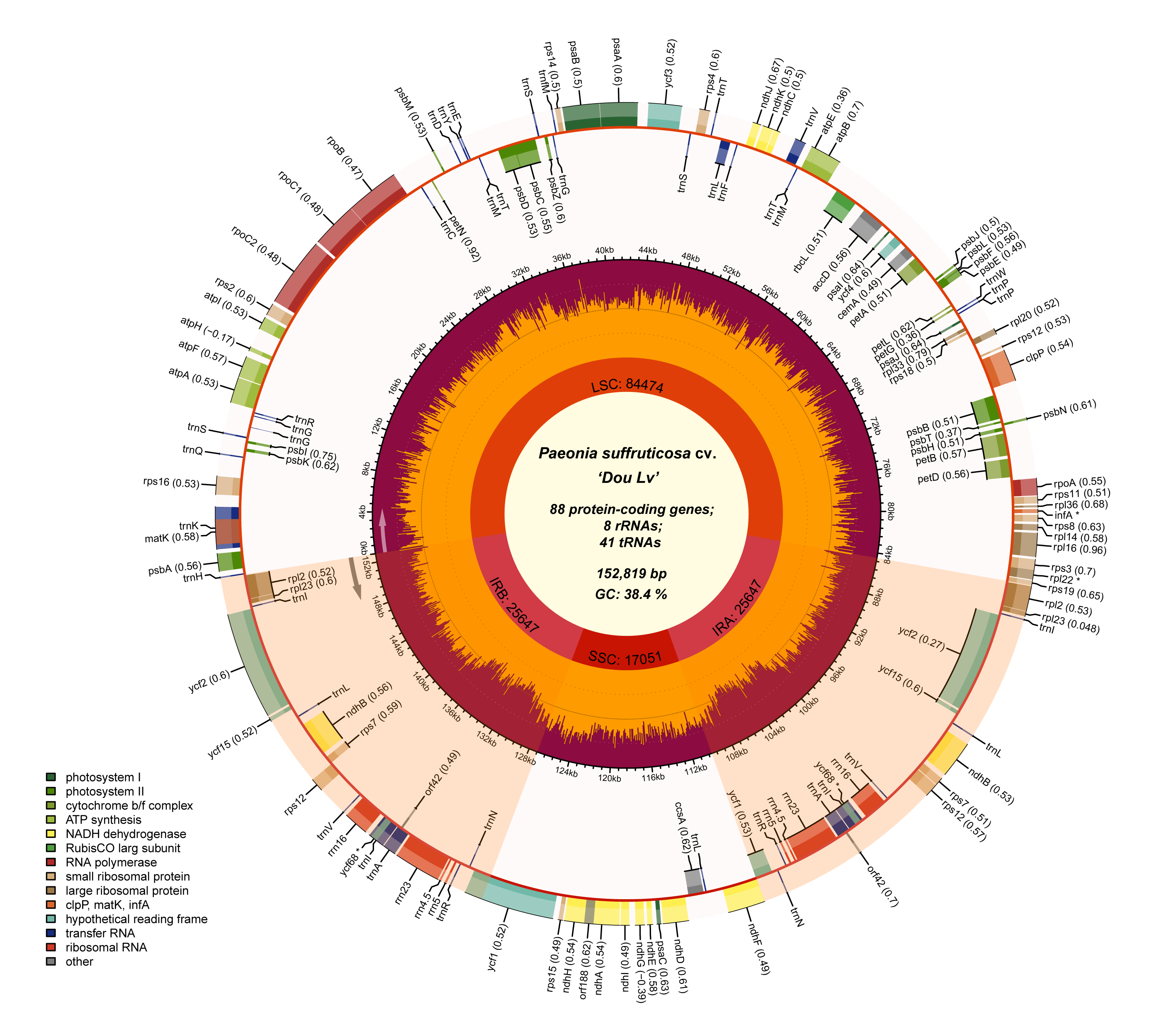

Supplement: Supplementary file 1 [file genes-13-02229-s001.zip › Supplementary Figure/Supplementary Figure S4.tif]

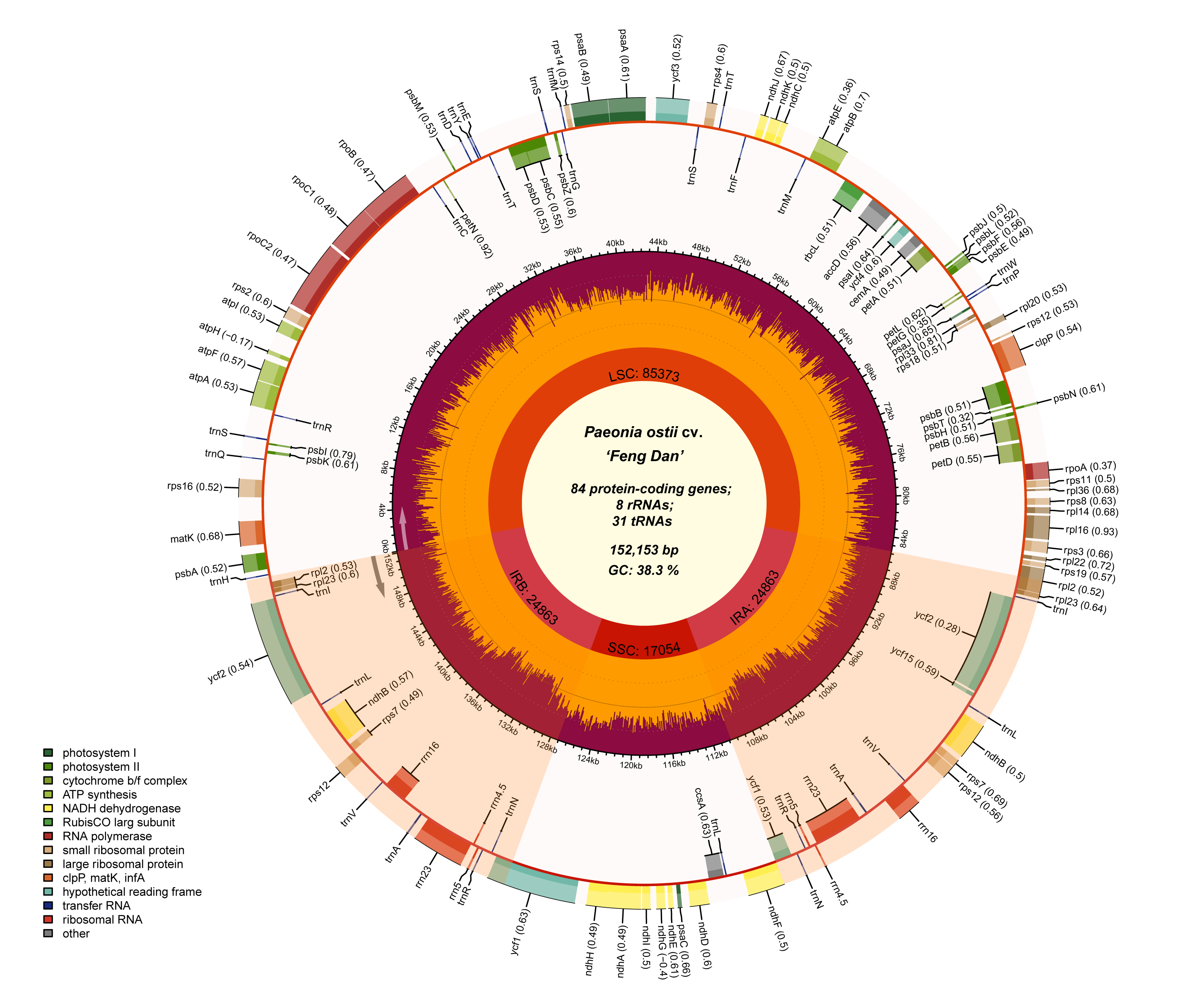

Supplement: Supplementary file 1 [file genes-13-02229-s001.zip › Supplementary Figure/Supplementary Figure S5.tif]

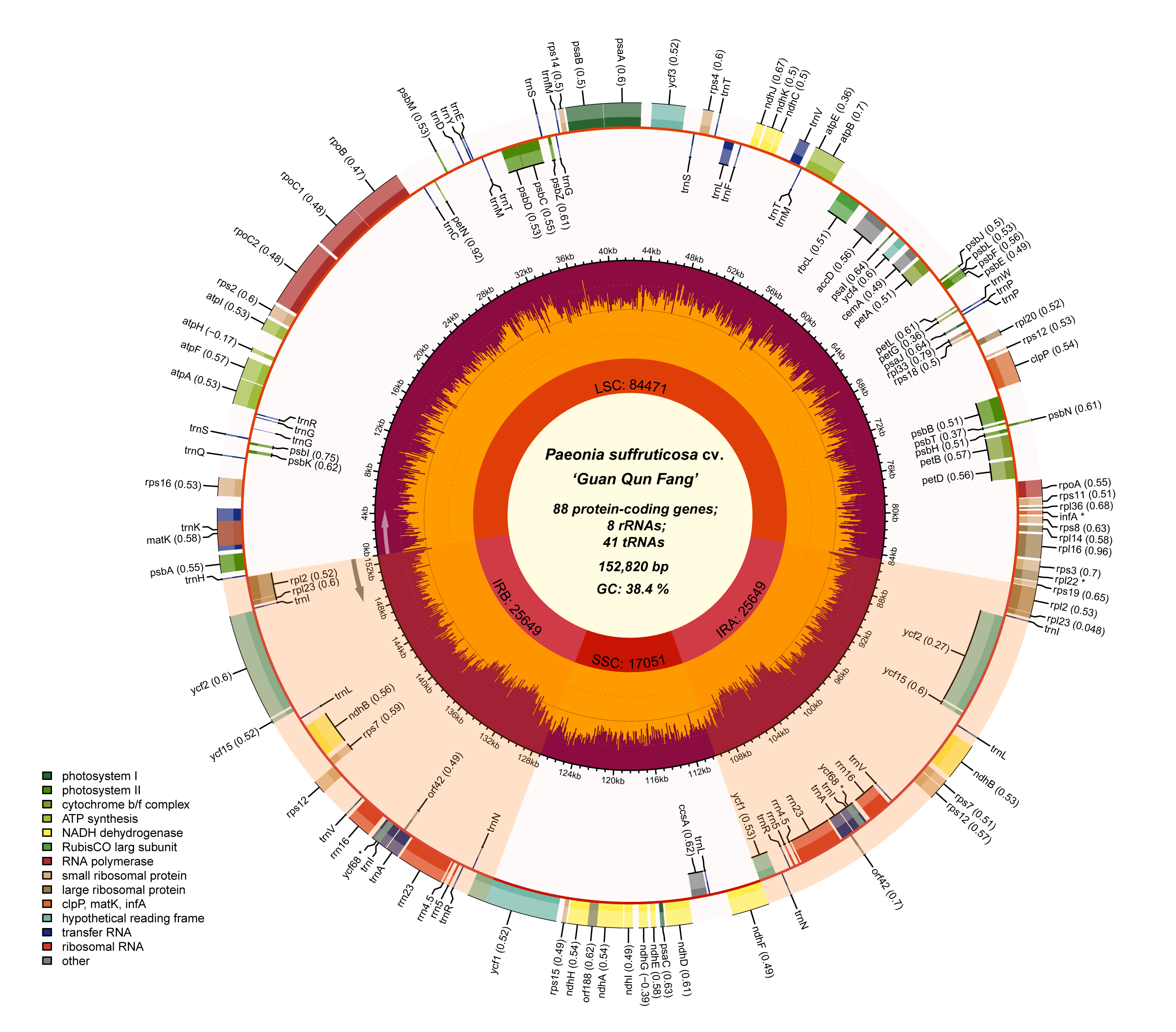

Supplement: Supplementary file 1 [file genes-13-02229-s001.zip › Supplementary Figure/Supplementary Figure S6.tif]

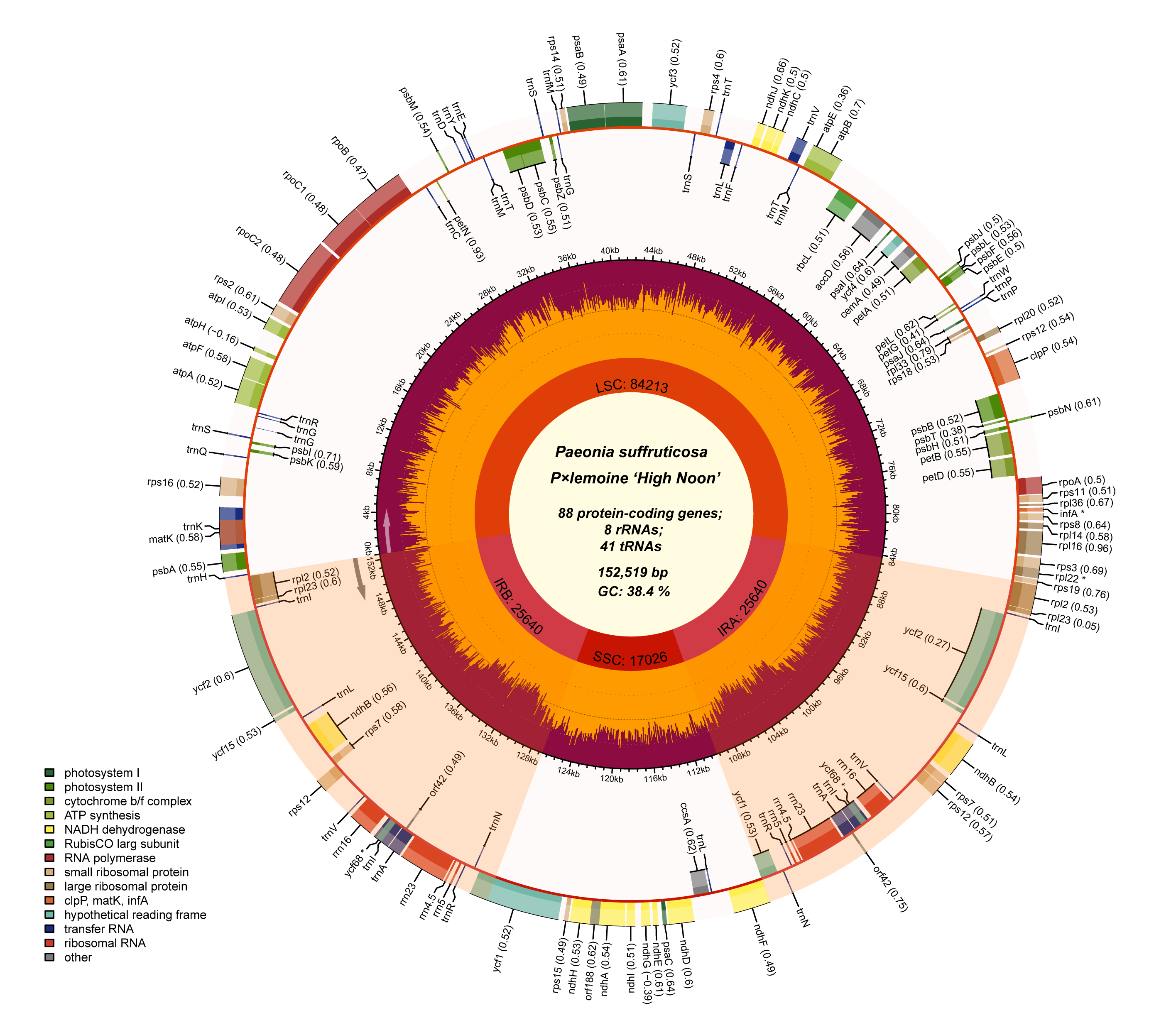

Supplement: Supplementary file 1 [file genes-13-02229-s001.zip › Supplementary Figure/Supplementary Figure S7.tif]

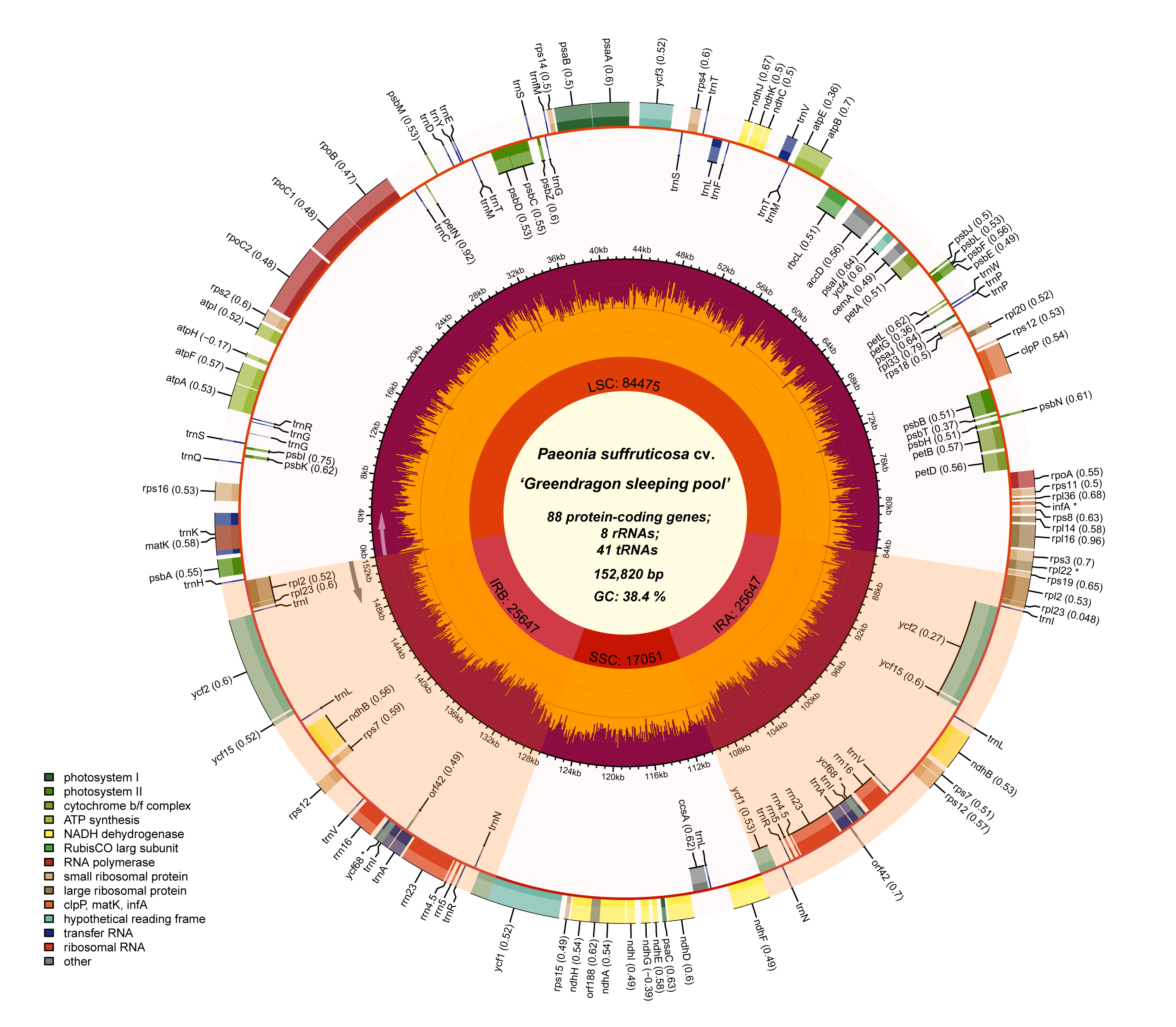

Supplement: Supplementary file 1 [file genes-13-02229-s001.zip › Supplementary Figure/Supplementary Figure S8.tif]

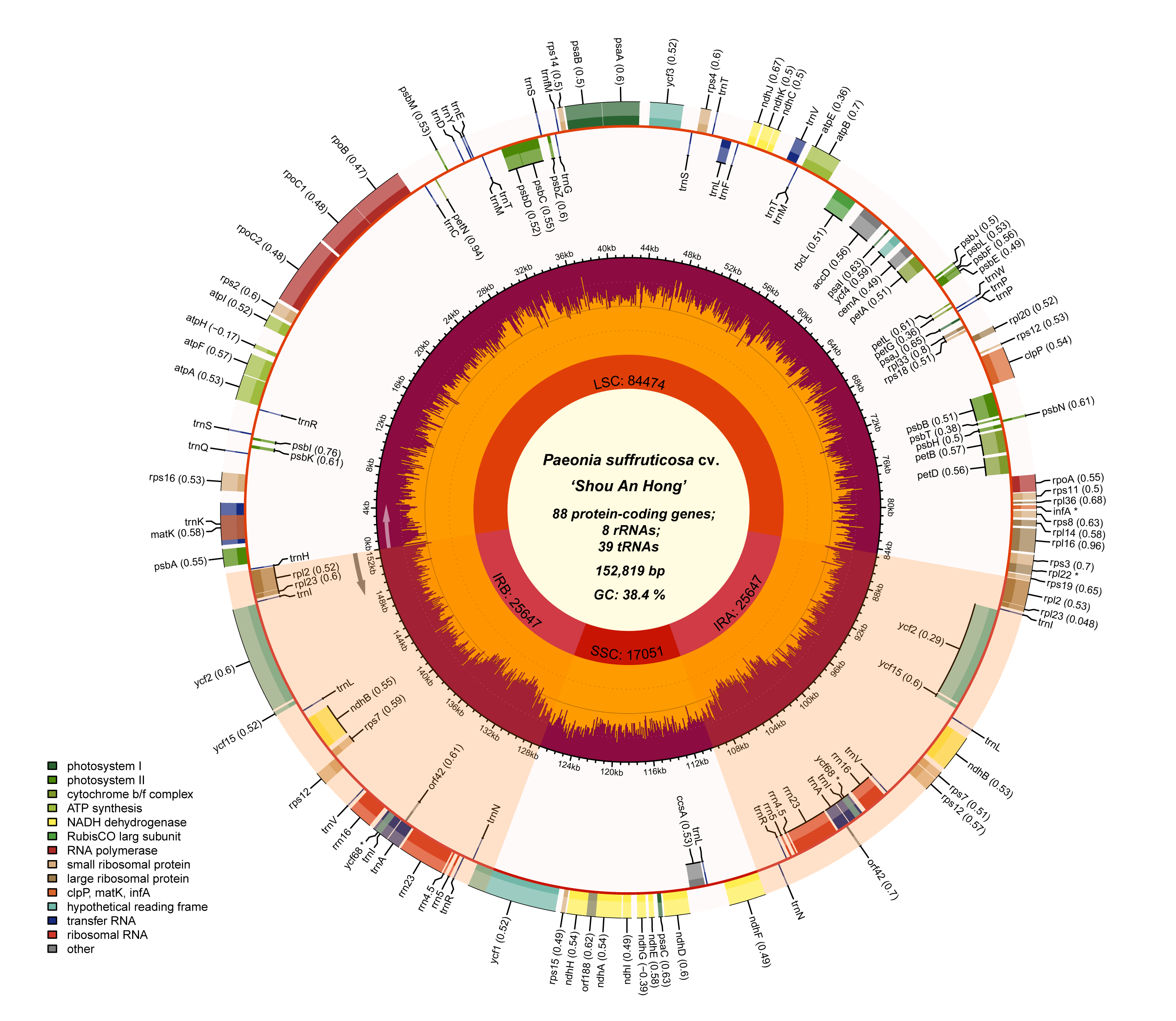

Supplement: Supplementary file 1 [file genes-13-02229-s001.zip › Supplementary Figure/Supplementary Figure S9.tif]
